# The cost‐effectiveness of universal late‐pregnancy screening for macrosomia in nulliparous women: a decision analysis

**DOI:** 10.1111/1471-0528.15809

**Published:** 2019-06-05

**Authors:** D Wastlund, AA Moraitis, JG Thornton, J Sanders, IR White, P Brocklehurst, GCS Smith, ECF Wilson

**Affiliations:** ^1^ The Primary Care Unit Department of Public Health and Primary Care University of Cambridge Cambridge UK; ^2^ Cambridge Centre for Health Services Research Cambridge Institute of Public Health University of Cambridge Cambridge UK; ^3^ Department of Obstetrics and Gynaecology NIHR Cambridge Biomedical Research Centre University of Cambridge Cambridge UK; ^4^ Division of Child Health, Obstetrics and Gynaecology School of Medicine University of Nottingham Nottingham UK; ^5^ School of Healthcare Sciences Cardiff University Cardiff UK; ^6^ MRC Clinical Trials Unit University College London London UK; ^7^ Birmingham Clinical Trials Unit University of Birmingham Birmingham UK; ^8^ Health Economics Group Norwich Medical School University of East Anglia Norwich UK

**Keywords:** Economic modelling, health economics, macrosomia, pregnancy, screening, third‐trimester, ultrasound

## Abstract

**Objective:**

To identify the most cost‐effective policy for detection and management of fetal macrosomia in late‐stage pregnancy.

**Design:**

Health economic simulation model.

**Setting:**

All English NHS antenatal services.

**Population:**

Nulliparous women in the third trimester treated within the UK NHS.

**Methods:**

A health economic simulation model was used to compare long‐term maternal–fetal health and cost outcomes for two detection strategies (universal ultrasound scanning at approximately 36 weeks of gestation versus selective ultrasound scanning), combined with three management strategies (planned caesarean section versus induction of labour versus expectant management) of suspected fetal macrosomia. Probabilities, costs and health outcomes were taken from literature.

**Main outcome measures:**

Expected costs to the NHS and quality‐adjusted life‐years (QALYs) gained from each strategy, calculation of net benefit and hence identification of most cost‐effective strategy.

**Results:**

Compared with selective ultrasound, universal ultrasound increased QALYs by 0.0038 (95% CI 0.0012–0.0076), but also costs by £123.50 (95% CI 99.6–149.9). Overall, the health gains were too small to justify the cost increase given current UK thresholds cost‐effective policy was selective ultrasound coupled with induction of labour where macrosomia was suspected.

**Conclusions:**

The most cost‐effective policy for detection and management of fetal macrosomia is selective ultrasound scanning coupled with induction of labour for all suspected cases of macrosomia. Universal ultrasound scanning for macrosomia in late‐stage pregnancy is not cost‐effective.

**Tweetable abstract:**

Universal late‐pregnancy ultrasound screening for fetal macrosomia is not warranted.

## Tweetable abstract

Universal late‐pregnancy ultrasound screening for fetal macrosomia is not warranted.

## Introduction

The detection and management of macrosomia, i.e. excessive fetal growth, poses a challenge to maternity care. Macrosomia is associated with increased perinatal mortality and morbidity, e.g. shoulder dystocia leading to brachial plexus injury, as well as increased risk of maternal morbidity.^1^, ^2^, ^3^ The definition of macrosomia varies, but is usually defined as a birthweight >4000 or >4500 g. It is differentiated from, but closely related to, the concept of large‐for‐gestational‐age, which is a relative measure: weight greater than the 90th centile for a given gestational age.^1^, ^4^ Macrosomia can only be definitively diagnosed by weighing the infant following delivery. However, ultrasound scans can be used to estimate the fetal weight antenatally, although this approach is known to have low predictive value.^1^ There is no general agreement on how to manage macrosomia if it is suspected following ultrasound.^1^, ^4^, ^5^, ^6^ Possible interventions include scheduling an elective Caesarean section (CS), or early induction of labour. However, uncertainty regarding the clinical effectiveness of these interventions persists.^1^, ^5^ Furthermore, if given without clinical need, intervention may cause unnecessary harm, e.g. neonatal respiratory morbidity, and the increased maternal risks of CS.^1^, ^4^, ^7^, ^8^

There is currently no national programme that couples screening for macrosomia with a proven, disease‐modifying intervention.^4^, ^9^ Currently, clinical examination of third‐trimester pregnancies does not routinely include ultrasound, but women may be selected for ultrasound scanning following clinical suspicion of macrosomia (selective ultrasound). An alternative approach would be to prospectively scan all women for macrosomia (universal ultrasound) at around 36 weeks of gestation, but whether the benefits of such an approach would justify the increased costs and risk of harmful interventions is unclear. A previous study showed only modest health benefits from universal ultrasound, and the cost for every prevented severe adverse outcome was too high to justify routine scanning.^10^ However, this study is now over 20 years old and only considered one management strategy for suspected macrosomia: delivery by planned CS. Following recent research and changes in obstetric care, we sought to re‐evaluate the case for universal ultrasound screening for macrosomia.^11^

In this study, we identify the most cost‐effective strategy for detection and management of macrosomia in late pregnancy among nulliparous women in the setting of the UK National Health Service (NHS).

## Methods

### Model structure

**Figure 1:**
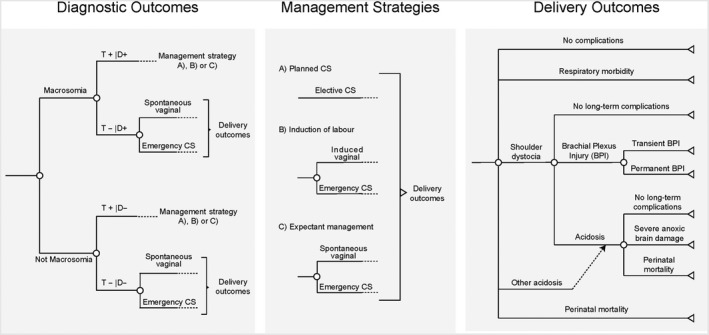
Structure of simulation model. The figure shows the model structure, from screening to long‐term health outcomes. Part A (left) shows the pathway from screening to the mode of delivery. When macrosomia is suspected (‘T+’), the mode of delivery depends on the management strategy as shown in part B (middle). Part C (right) shows the different delivery outcomes, and their associated long‐term outcomes. BPI, brachial plexus injury; D+, disease‐positive; D−, disease‐negative; T+, test‐positive; T−, test‐negative.

The scope of this model was limited to screening for macrosomia rather than any other complication of pregnancy. To compare the cost‐effectiveness of different policies for detection and management, we constructed a decision tree simulation model using R (Figure 1).^12^, ^13^, ^14^ Each policy had two components: one for the detection of macrosomia, and one for the management of suspected macrosomia. The detection strategy was either universal ultrasound in the third trimester (around 36 weeks of gestation), or selective ultrasound, i.e. clinical examination through abdominal palpation, where ultrasound would be offered only where macrosomia was suspected. The management strategy for suspected macrosomia was either to schedule an elective CS (Planned CS), induce labour (Induction), or expectant management awaiting spontaneous labour onset. If macrosomia was not suspected, expectant management was used. There are therefore a total of six discrete detection/management policies.

The model structure for detection and management for macrosomia is shown in Figure 1(A). Four different screening statuses were possible: true positives, false negatives, false positives and true negatives. The likelihood of each state was driven by the sensitivity and specificity of the test used for detection, as well as the prevalence of macrosomia. When macrosomia was suspected, the pregnancy was managed according to the management strategy being evaluated: planned CS, induction of labour, or expectant management. If macrosomia was not suspected, it was assumed that vaginal delivery would be attempted, with a risk of emergency CS. To accurately capture the consequences of a false‐positive diagnosis of macrosomia, we distinguished between expectant management when macrosomia was suspected or not suspected; suspected macrosomia increased the risk of Caesarean delivery following expectant management.^8^

Five neonatal delivery outcomes were possible: No complications, Respiratory morbidity, Shoulder dystocia, Other acidosis (i.e. acidosis not induced by shoulder dystocia) and perinatal mortality. Their respective likelihoods were affected by both screening and management strategies (see below). The fetal delivery outcomes were then extrapolated into long‐term costs and quality‐adjusted life‐years (QALYs) through the model shown in Figure 1.

### Model inputs

#### Probabilities

For each adverse outcome (respiratory morbidity, shoulder dystocia, other acidosis and mortality), we obtained the baseline risk of that outcome; i.e. the risk if infant was a non‐large and non‐induced neonate with vaginal delivery. We then multiplied this risk with the relative risk of each present risk factor (macrosomia, induction, delivery through elective CS and delivery through emergency CS). For technical details, see Supplementary material (Appendix S1).

Model input parameters are shown in the Supplementary material (Table S1). Values were identified from literature by AM and DW, prioritising values from systematic reviews and UK data where possible. Ideally, every input should be based upon a systematic review, reflecting current state of knowledge. However, resources only permitted identification of suitable data, rather than performing a meta‐analysis. For this reason, sources that provided a distribution for the likely parameter values were prioritised, so that the overall uncertainty associated with this parameter could be assessed through probabilistic sensitivity analysis.^15^ Where multiple sources were available the source was chosen by consensus or through arbitration by GS. Where no credible values for a model parameter could be identified from the literature, AM and GS identified lower and upper limits to the value that the parameter could reasonably assume; the model then sampled input values from this interval using a uniform distribution.

Macrosomia was defined as estimated fetal weight ≥90th centile, i.e. the same as large‐for‐gestational‐age. The sensitivity and specificity for detection of macrosomia, as well as the prevalence of macrosomia, were taken from the POP study, a prospective cohort study of unselected nulliparous women in which all women had fetal biometry at 36 weeks of gestation, where the result of the scan was blinded.^16^, ^17^ Using data from this study allowed for a comparison between diagnostic performance of universal and selective ultrasound. Detection with selective ultrasound was based upon clinical suspicion before 36 weeks of gestation following measurement of symphyseal–fundal height, and confirmed with a clinically indicated ultrasound.^17^ The baseline risk of each adverse outcome was defined as the risk for a normal‐size neonate, where labour was not induced and resulted in a vaginal delivery. We used odds ratios from the literature when directly presented, otherwise we calculated unadjusted odds ratios from prevalence data.^18^ Odds ratios were assumed to be log‐normally distributed.

#### Long‐term outcomes

Unit costs and health state utilities are shown in the Supplementary material (Table S1). The average costs for induction of labour and respiratory morbidity were calculated from the NHS reference costs (see Supplementary material, Appendix S2).^19^ Brachial plexus injury could be either transient or permanent, this was modelled using a β distribution.^20^ We assumed that brachial plexus injury would require the same resource usage as reported by Culligan et al., and obtained the costs for these resources from the NHS reference costs (see Supplementary material, Appendix S2).^19^, ^21^ We assumed that all cases of nonsevere asphyxia would be treated in the neonatal unit for 1–3 days, but that no additional costs would accrue beyond this. To estimate the long‐term outcomes from ‘severe anoxic brain damage’, we made the simplifying assumption that the costs, consequences and likelihood mirrored those of neonatal encephalopathy. Evidence shows that providing therapeutic hypothermia reduces the likelihood of adverse outcomes from neonatal encephalopathy, and this treatment is routine clinical practice.^22^, ^23^ We assumed that all cases of neonatal encephalopathy would receive therapeutic hypothermia, and adjusted costs and consequences from neonatal encephalopathy accordingly; for this reason, we reduced the likelihood of mortality and severe anoxic brain damage following asphyxia by 11.1%.^24^ The costs from severe anoxic brain damage included hospital‐ and community‐care costs for all survivors in the cooled group as reported by Regier et al.;^22^ the hospital costs were for the first 18 months only, but we assumed that the community‐care costs after discharge would accrue annually for the entirety of the model's time horizon. We made the simplifying assumption that the cost of death would be the same regardless of reason.

Quality‐adjusted life‐years combine the utility of a health‐state with its duration, where utility is based upon quality of life (QOL). Quality of life can be expressed as a numeric value, where 1 is equivalent to full health and 0 is equivalent to death.^25^, ^26^ Maternal QALYs were based upon the mode of delivery, and QOL weights were obtained from Petrou et al.;^27^ these QOL weights were derived using EQ‐5D, as recommended by NICE.^28^, ^29^ For surviving infants, we calculated the expected QALYs based upon the assumptions above; per definition, fetal QALYs were zero for death.

### Model scope

The expected cost and QALYs gained from six different policies for screening and management of macrosomia were calculated over a 20‐year time horizon. Costs and QALYs were discounted by 3.5% annually, as recommended by NICE.^29^ Probabilistic sensitivity analysis was used to capture the overall effect of uncertainty in the model parameters. Costs associated with potential litigation claims or potential effects upon subsequent pregnancies were not included. Results were based upon 100 000 simulations and results presented as expected values, incremental cost and QALYs, incremental cost‐effectiveness ratios (ICERs) (the ratio of incremental cost to incremental QALYs), and net benefits (defined as QALYs multiplied by the willingness to pay [WTP] for a QALY less the cost). The WTP per QALY threshold was assumed to be £20,000 (the lower of NICE's stated thresholds).^29^ Decision uncertainty is illustrated using cost‐effectiveness acceptability curves.^29^, ^30^ The model's sensitivity towards key parameters was explored through one‐way sensitivity analysis (see Supplementary material, Appendix S4). Given the paucity of data relating to maternal quality of life, an additional scenario was conducted including neonatal QALYs alone. Further scenarios explored the impact of assigning zero additional costs for induction of labour, and assuming that induction of labour is cost saving (due to reduced antenatal assessments).^29^, ^30^ All costs are from the third‐party payer (i.e. NHS) perspective, and the price year is 2016/17. Costs from other years were inflated to the price year of the analysis using the Hospital & Community Health Services index.^31^ As this is a secondary analysis/synthesis of existing data, no patients nor the public were involved in the study.

## Results

The expected costs and QALYs for each policy are shown in Table 1. The least expensive option is selective ultrasound with expectant management and the most expensive option is universal ultrasound with planned CS. The least effective option (in terms of QALYs gained) is universal ultrasound with planned CS and the most effective option is universal ultrasound with induction of labour. Three strategies (selective US + planned CS, universal ultrasound + expectant management, and universal ultrasound + planned CS) are dominated or extended‐dominated by other strategies. Taking into account the balance between costs and outcomes (and with a WTP threshold of £20,000 per QALY), the most cost‐effective strategy is selective ultrasound plus induction of labour where macrosomia is suspected. Although universal ultrasound plus induction is expected to yield marginally greater QALYs (+0.002), the added cost (+£113) yields an ICER of £52,719. This is above the threshold and is not, therefore, cost‐effective. The expected distribution of mode of delivery and neonatal delivery outcomes is detailed in the Supplementary material (Appendix S3 and Table S2).

**Table 1:** Expected costs and QALYs per screening and management strategy

Strategy	Cost (95% CI)	QALY (95% CI)^a^	ICER	NMB (95% CI)
Selective ultrasound + expectant	2821 (2409–3236)	27.441 (27.262–27.621)	—	546 007 (542 803–549 204)
**Selective ultrasound + induction**	**2826 (2412–3242)**	**27.446 (27.267–27.626)**	**904**	**546 098 (542 890–549 298)**
Selective ultrasound + planned CS	2833 (2436–3230)	27.417 (27.244–27.588)	Dominated	545 501 (542 424–548 561)
Universal ultrasound + expectant	2933 (2502–3366)	27.441 (27.261–27.621)	Dominated	545 884 (542 695–549 070)
Universal ultrasound + induction	2939 (2506–3374)	27.448 (27.268–27.628)	52 719	546 028 (542 829–549 214)
Universal ultrasound + planned CS	2955 (2549–3360)	27.396 (27.224–27.565)	Dominated	544 956 (541 919–547 978)

NMB, net monetary benefit.

Options ordered from lowest to highest expected cost. ICERs calculated beginning with least expensive option, and comparing with next most expensive, non‐dominated option; a policy was dominated/extended‐dominated if any other policy or weighted average of two policies was associated with both lower costs and higher QALYs. Net monetary benefit (NMB) was calculated using a WTP threshold of £20,000; higher NMB value means greater cost‐effectiveness. Option with the highest expected net monetary benefit highlighted in bold. All costs and NMB are given in pounds sterling (£).

a The maximum QALYs for two people over 20 years, discounted at 3.5%, is 29.42.

We investigated the value of universal ultrasound alone by comparing the results for universal and selective ultrasound when using the same management strategy. When the management strategy was planned CS, universal ultrasound was associated with a cost increase of £123.50 (95% CI £99.60–£149.90), and a QALY increase of 0.0038 (95% CI 0.0012–0.0076). The ICER for this strategy was £35,755 (95% CI £15,962–£98,506). The comparable ICERs for induction of labour and expectant management were even higher, indicating that universal ultrasound screening is unlikely to be cost‐effective.

**Figure 2:**
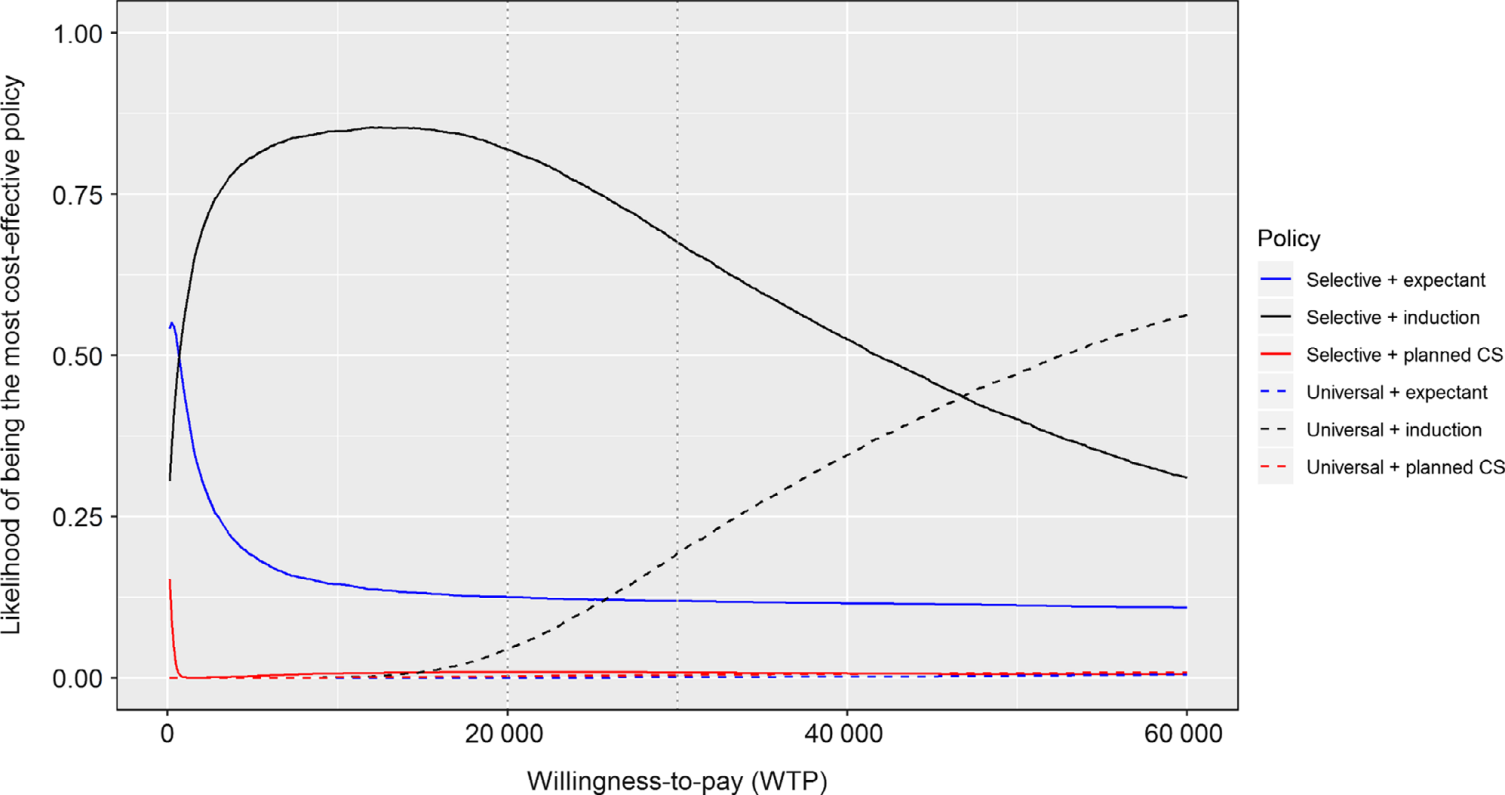
Cost‐effectiveness acceptability curve for policies for detection and management of fetal macrosomia. Cost‐effectiveness acceptability curve showing the chance of each policy of being the most cost‐effective for different levels of WTP. Policies with universal ultrasound are shown as dashed lines and selective ultrasound as solid. Higher values for WTP imply a higher valuation of a QALY. The conventional WTP threshold for cost‐effectiveness is £20,000–£30,000 (marked in figure).^29^

The probability of each policy being the most cost‐effective as a function of the WTP threshold is shown by the cost‐effectiveness acceptability curves (Figure 2). Selective ultrasound coupled with induction of labour for suspected macrosomia had the greatest chance of being cost‐effective for NICE's recommended thresholds of £20,000–£30,000 per QALY.^29^ Sensitivity analysis showed that the choice of policy was most sensitive towards the specificity of ultrasound (both universal and selective), maternal QOL for delivery through elective CS, and the prevalence of macrosomia (see Supplementary material, Appendix S4 and Table S3). Although influential, the cost of ultrasound screening alone appears insufficient to determine whether universal screening would be cost‐effective; analysis showed that if other parameters remained unchanged, universal ultrasound would only be cost‐effective if the cost of ultrasound was £26.56 or lower.

Excluding maternal QALYs from the analysis, selective ultrasound plus planned CS was the preferred management strategy, compared with induction of labour, under the base case (see Supplementary material, Table S4). No other assumptions tested in the alternative scenarios affected the conclusions; selective ultrasound with induction of labour remained the preferred strategy for all other scenarios.

## Discussion

### Main findings

This study has compared the cost‐effectiveness of different policies for detection and management of fetal macrosomia in late‐stage pregnancy among nulliparous women. The most cost‐effective policy was selective ultrasound coupled with induction of labour for all cases of suspected fetal macrosomia. Although universal ultrasound scanning leads to higher identification of suspected macrosomia, this only translates into modest improvements of overall long‐term health outcomes, which are not justified by the added cost of the ultrasound scan. The expected health gain (0.003 QALYs over 20 years) is small because of both the low risk of severe neonatal outcomes resulting from undiagnosed macrosomia and the risk of interventions themselves causing harm.

Where macrosomia is suspected following ultrasound scanning, intervention is generally preferred to awaiting spontaneous labour onset. Although currently subject to further research,^32^ this study found that induction of labour is the preferred intervention. However, it is worth noting that from the infant's perspective alone, the best option is an elective CS (see Supplementary material, Table S4, scenario ‘Maternal QALYs excluded’).

Universal (rather than selective) ultrasound coupled with induction of labour has the potential to be the most cost‐effective policy, but only at very high valuations of health gain: the small added benefit does not currently justify the cost. Sensitivity analysis shows that the relative cost‐effectiveness of the policies is sensitive to changes in the cost of ultrasound scanning, as well as the costs of CS and induction of labour, and the sensitivity and specificity of ultrasound scanning. Hence, if the cost of the scan falls substantially in the future, a universal scanning policy could be cost‐effective; analysis shows that this would happen at a cost below £26.56 (a cost reduction of 74.4%). Further, macrosomia is not the only fetal complication that can be assessed through ultrasound screening, so when combined with a scan for other anomalies, such as breech presentation, the marginal cost of detecting macrosomia may be sufficiently low to render the overall policy cost‐effective. However, further work is needed to explore this.

### Strengths and limitations

The strength of this study is that it evaluates strategies for both detection and management of fetal macrosomia jointly. There has been a lack of studies evaluating screening strategies coupled with clear evidence‐based interventions. Economic modelling allows us to estimate how neonatal and maternal health outcomes would be affected if ultrasound screening were to be routinely implemented in clinical practice. However, the robustness of the conclusions is only as strong as the data available to inform them. Indeed, many parameters were informed by a single study, and where no data were available we relied on expert opinion. Critically, as a part of this process we elicited a range of plausible values to represent the inherent uncertainty. The probabilistic sensitivity analysis incorporates this uncertainty to determine how much it affects the overall results.

We have limited our analysis to nulliparous women. It is unclear whether our findings could be extended to parous women as well, especially given the absence of data on screening performance for universal and selective ultrasound for this group. The economic modelling also relies upon simplifying assumptions regarding the long‐term outcomes from the mode of delivery and fetal delivery outcomes and did not take account of alterations to planned place of birth following ultrasound. The interplay between fetal macrosomia and long‐term outcomes may be too complex to capture entirely within our model; macrosomia can lead to more complications than those explored in this analysis. However, in the absence of more detailed data on many of these complications, this model is still based upon the best current understanding of macrosomia and its consequences.

The probability of delivery outcomes in this analysis relied upon the assumption of no interaction between macrosomia and the intervention. In reality, this assumption may not hold perfectly; for example, elective CS may yield a greater relative risk reduction for babies with macrosomia. However, data limitations made the assumption necessary in order to model the relevant outcomes, especially given the many different sources used for parameters. Also, the relative risks associated with both macrosomia and interventions were included in the analysis, even though interactions were not modelled.

### Interpretations

Our conclusion that universal ultrasound screening for fetal macrosomia is not cost‐effective aligns with previous findings for macrosomia management based upon ultrasound screening.^10^ Universal ultrasound screening strategies were less cost‐effective than selective ultrasound for all scenarios. Our analysis demonstrated that universal ultrasound is associated with improved health outcomes, but that these gains are too small to justify its added cost.

This analysis is based in a UK NHS setting. The results will be generalisable to other settings with similar management policies and relative costs: current UK practice is to offer a scan at first and second trimesters but to only offer late‐pregnancy scans where clinically indicated (our ‘selective ultrasound’ policy). Many European countries perform a third scan around 32 weeks.^33^ Diagnostic effectiveness at 32 weeks for predicting complications related to macrosomia at delivery is likely to be poorer than at the 36–37 weeks assumed in our analysis, given the longer interval between the scan and time of birth.^16^ This would suggest that earlier scans are even less likely to be cost‐effective.

As stated above, the impact of CS on maternal QOL was a key driver of the results. To the best of our knowledge, the study by Petrou et al.^27^ is the only study that reports maternal QOL as a function of the mode of delivery, using an adequate time horizon and a measure for QOL recommended by NICE.^29^ However, it reported lower QOL for women who underwent elective CS than their counterparts who delivered through emergency CS, a finding that appears counterintuitive. If maternal QOL had been higher following elective CS than emergency CS, the economic analysis would have been more favourable towards policies with planned CS. Against this should be weighted the research that has shown that CS is associated with increased risk of a range of complications in subsequent pregnancies.^34^, ^35^, ^36^ These risks are not captured in our simulation model because the perspective was for the current pregnancy, but implies that managing suspected macrosomia through planned CS may be more detrimental than suggested in this analysis.

This analysis has compared interventions based upon suspicion of macrosomia alone. However, in clinical practice more factors influence antenatal management than just whether ultrasound screening indicates fetal macrosomia. This analysis offers valuable information for policymaking, but it does not rule out the use of planned CS or expectant management in individual cases.

## Conclusion

Universal ultrasound scanning in the third trimester is not cost‐effective at detecting macrosomia in nulliparous women at current UK cost‐effectiveness threshold limits. If fetal macrosomia is suspected following ultrasound, induction of labour is likely to be the most cost‐effective management option.

The conclusions are based on a single scan for macrosomia alone. A strategy that combines scanning for macrosomia with other conditions, e.g. breech presentation (and growth restriction), might be cost‐effective. Future research should focus on whether joint screening for multiple fetal complications would be cost‐effective, as well as on the long‐term health consequences from delivery outcomes, especially how maternal health is affected by the mode of delivery.

### Disclosure of interests

PB reports grants and personal fees from MRC, grants from MRC, NIHR HS&DR, NIHR HTA, Wellcome Trust, and personal fees from AG Biotest, outside the submitted work. GS reports grants and personal fees from GlaxoSmithKline Research and Development Limited, grants from Sera Prognostics Inc., grants and personal fees from Roche Diagnostics Ltd, and non‐financial support from Illumina Inc., outside the submitted work. In addition, GS has a patent pending: United Kingdom Patent Application No. 1808489.7 ‘Novel Biomarkers’. AM, DW, EW, IRW, JS and JGT declare no competing interests. Completed disclosure of interest forms are available to view online as supporting information.

### Contribution to authorship

AM, DW, EW and GS contributed to the study concept. Data collection was by AM and DW, and economic analysis by DW and EW. AM, DW, EW, GS, IRW, JS, JGT and PB all contributed to the manuscript preparation.

### Details of ethics approval

Not applicable.

### Funding

This study was funded by the National Institute for Health Research (NIHR) Health Technology Assessment programme, grant number 15/105/01. EW is part funded by the NIHR Cambridge Biomedical Research Centre. IRW was supported by the Medical Research Council Programme MC_UU_12023/21. The views expressed here are those of the authors and not necessarily those of the NHS, the NIHR or the Department of Health.

## Supporting information


**Table S1.** Input values for simulated model.

**Table S2.** Expected share of mode of delivery and level of fetal complications per screening and management strategy.

**Table S3.** Sensitivity of net monetary benefit towards input parameters.

**Table S4.** Expected costs and QALYs per screening and management strategy, alternative scenarios.

**Appendix S1.** The risk of adverse neonatal delivery outcomes.
**Appendix S2.** Detailed derivation of model inputs.
**Appendix S3.** Expected distribution of outcomes.
**Appendix S4.** Sensitivity of results.■
