# Supplementary material for: The cost‐effectiveness of universal late‐pregnancy screening for macrosomia in nulliparous women: a decision analysis
Source: BJOG. 2019 Jun 5;126(10):1243–50. doi: 10.1111/1471-0528.15809 (PMC6771727; doi:10.1111/1471-0528.15809)
Supplement: Supplementary file 1 — Table S1. Input values for simulated model. [file BJO-126-1243-s001.pdf]

Table S1. Input values for simulated model

Parameter	Mean	95% CI	Distribution and inputs	Source
**Screening probabilities**				
Macrosomia prevalence	0.1107	0.1008, 0.1206	~Normal(0.1107, 0.005)	Sovio et al.[10]
Sensitivity 'Universal ultrasound'	0.3785	0.3087, 0.4510	~Beta(67, 110)	Sovio et al.[10]
Specificity 'Universal ultrasound'	0.9656	0.9595, 0.9712	~Beta(3562, 127)	Sovio et al.[10]
Sensitivity 'Selective ultrasound'	0.2655	0.2033, 0.3328	~Beta(47, 130)	Sovio et al.[10]
Specificity 'Selective ultrasound'	0.9867	0.9828, 0.9902	~Beta(3640, 49)	Sovio et al.[10]
**Management probabilities**				
Risk of emergency CS, induced	0.1632	0.1520, 0.1744	~Normal(0.1632, 0.0057)	Middleton et al.[11]
Risk of emergency CS, expectant	0.1842	0.1771, 0.2017	~Normal(0.1842, 0.0063)	Middleton et al.[11]
RR of emergency CS, macrosomia suspected	1.5165	0.9899, 2.3232	~Log-normal(0.4164, 0.2176)	Blackwell et al.[12]
**Long-term probabilities**				
NICU rate from respiratory morbidity	0.175	0.056, 0.294	~Uniform(0.05, 0.30)	Expert opinion
BPI risk if shoulder dystocia	0.0856	0.0614, 0.1098	~Normal(0.0856, 0.0123)	MacKenzie et al.[13]
Anoxia risk if shoulder dystocia	0.07	0.0480, 0.0921	~Normal(0.0700, 0.0113)	MacKenzie et al.[13]
Risk of permanent BPI	0.075	0.051, 0.988	~Uniform(0.05, 0.10)	RCOG GTG No. 42[14]
Risk of severe anoxic brain damage †	0.1527	0.1145, 0.1908	~Normal(0.1527, 0.0195)	Graham et al.[15]
Mortality from anoxia †	0.0524	0.0321, 0.0728	~Normal(0.0524, 0.0104)	Graham et al.[15]
**Neonatal delivery probabilities**				
Respiratory morbidity (baseline)	0.0032	0.0018, 0.0046	~Normal(0.0032, 0.0007)	Morrison et al.[1]
RR of respiratory morbidity, macrosomia	0.75	0.5125, 0.9875	~Uniform(0.50, 1.00)	Expert opinion
RR of respiratory morbidity, induced	0.5654	0.3900, 0.8196	~Log-normal(-0.5703, 0.1892)	Gibson et al.[16]
RR of respiratory morbidity, elective CS	5.7182	2.6190, 12.4850	~Log-normal(1.7437, 0.3984)	Morrison et al.[1]
RR of respiratory morbidity, emergency CS	5.1946	2.0934, 12.8900	~Log-normal(1.6476, 0.4637)	Morrison et al.[1]
Shoulder dystocia (baseline)	0.64	0.58, 0.97	~Uniform(0.58, 0.70)	RCOG GTG No. 42[14]
RR of shoulder dystocia, macrosomia	7.7631	4.5956, 13.1143	~Log-normal(2.0494, 0.2675)	Rossi et al.[17]
RR of shoulder dystocia, induced	0.5781	0.3447, 0.9697	~Log-normal(-0.781, 0.2638)	Boulvain et al.[18]
RR of shoulder dystocia, elective CS	0	N/A	N/A	Assumption
RR of shoulder dystocia, emergency CS	0	N/A	N/A	Assumption
Other acidosis (baseline)	0.0068	0.0022, 0.0140	~Beta(5, 726)	Middleton et al.[11]
RR of other acidosis, macrosomia	5.7953	3.0198, 11.1228	~Log-normal(1.7571, 0.3326)	Rossi et al.[17]

RR of other acidosis, induced	1.8201	0.6070, 5.4580	~Log-normal(0.5989, 0.5603)	Middleton et al.[11]
RR of other acidosis, elective CS	0.3014	0.1088, 0.8347	~Log-normal(-1.1993, 0.5197)	Chongsuvivatwong et al.[19]
RR of other acidosis, emergency CS	1.8674	1.2173, 2.8647	~Log-normal(0.6245, 0.2183)	Chongsuvivatwong et al.[19]
Mortality (baseline)	0.0038	0.0036, 0.0040	~Normal(0.0038, 0.0001)	ONS[20]
RR of mortality, macrosomia	3.3494	1.7057, 6.5773	~Log-normal(1.2088, 0.3443)	Rossi et al.[17]
RR of mortality, induced	0.1242	0.0286, 0.5407	~Log-normal(-2.0855, 0.7503)	Middleton et al.[11]
RR of mortality, elective CS	0.31	0.14, 0.67	~Log-normal(-1.1712, 0.4003)	Chongsuvivatwong et al.[19]
RR of mortality, emergency CS	1.68	1.19, 2.37	~Log-normal(0.5934, 0.3445)	Chongsuvivatwong et al.[19]
**Cost (£)**				
Ultrasound cost	107.54	86.37, 128.71	~Normal(107.54, 10.8)	NHS Reference costs 2016-17[2]
Delivery cost, vaginal	2478.4	1992.3, 2964.5	~Normal(2478.4, 248))	NHS Reference costs 2016-17[2]
Delivery cost, elective CS	3555.8	2858.0, 4253.6	~Normal(3555.8, 356)	NHS Reference costs 2016-17[2]
Delivery cost, emergency CS	4774.7	3839.8, 5709.6	~Normal(4774.7, 477)	NHS Reference costs 2016-17[2]
Respiratory morbidity ††	2010	N/A	Point estimate	Morrison et al.[1]
Induction cost ††	278.5	220.1, 336.9	~Uniform(217, 340)	NHS Reference costs 2016-17[2]
Mortality cost	1623	1305, 1941	~Normal(1623, 162)	Regier et al.[21]
Transient BPI ††	2066	N/A	Point estimate	Culligan et al.[4]
Permanent BPI ††	14133	N/A	Point estimate	Culligan et al.[4]
Non-severe anoxia	2590	1360, 3820	~Uniform(1295, 3885)	Expert opinion
Severe anoxic brain damage	18632	N/A	Point estimate	Regier et al.[21]
**Neonatal utility**				
BPI - short-term	0.95	0.903, 0.998	~Uniform(0.90, 1.00)	Culligan et al.[4]
BPI - long-term	0.6	0.505, 0.695	~Uniform(0.50, 0.70)	Culligan et al.[4]
Anoxia - short-term	0.975	0.951, 0.999	~Uniform(0.95, 1.00)	Expert opinion
Severe anoxic brain damage	0.63	0.616, 0.644	~Normal(0.63, 0.007)	Leigh et al.[22]
**Maternal utility**				
Delivery - Vaginal	0.939	0.925, 0.953	~Normal(0.939, 0.007)	Petrou et al.[23]
Delivery – ELCS	0.882	0.839, 0.925	~Normal(0.882, 0.022)	Petrou et al.[23]
Delivery – EMCS	0.935	0.904, 0.966	~Normal(0.935, 0.016)	Petrou et al.[23]

Input values for the simulation model (see Fig 1). All prices are updated to the price level of 2016/17 using the hospital & community health services (HCHS) index.[6]
BPI = Brachial Plexus Injury; CI = Confidence interval; CS = Caesarean section; IOL = Induction of labour; NICU = Neonatal intensive care unit; RR = Relative risk.
† The mean and standard deviation has been lowered by 11.1% from the original source, following neonatal cooling, in accordance with Edwards et al.[24]
†† For details on calculations, see Appendix S2.
