# Supplementary material for: The cost‐effectiveness of universal late‐pregnancy screening for macrosomia in nulliparous women: a decision analysis
Source: BJOG. 2019 Jun 5;126(10):1243–50. doi: 10.1111/1471-0528.15809 (PMC6771727; doi:10.1111/1471-0528.15809)
Supplement: Supplementary file 3 — Table S3. Sensitivity of net monetary benefit towards input parameters. [file BJO-126-1243-s003.pdf]

Table S2. Expected share of mode of delivery and level of fetal complications per screening and management strategy

Strategy	Mode of delivery			Fetal delivery outcome				
	Vaginal	Elective CS	Emergency CS	No comp	RM	SD	Acidosis	Death
Universal US + planned CS	75.7%	7.3%	17.1%	97.025%	0.669%	0.724%	1.070%	0.511%
Universal US + induction	81.7%	0.0%	18.3%	96.678%	0.565%	0.836%	1.420%	0.501%
Universal US + expectant	80.8%	0.0%	19.2%	96.713%	0.592%	0.885%	1.237%	0.573%
Selective US + planned CS	78.2%	4.1%	17.7%	96.934%	0.630%	0.784%	1.121%	0.531%
Selective US + induction	81.7%	0.0%	18.3%	96.691%	0.573%	0.859%	1.353%	0.524%
Selective US + expectant	81.2%	0.0%	18.8%	96.717%	0.588%	0.893%	1.232%	0.571%

US = Ultrasound scan; CS = Caesarean section; No comp = No complications; RM = Respiratory morbidity; SD = Shoulder dystocia; Acidosis = Other acidosis (acidosis unrelated to shoulder dystocia).
