# Supplementary material for: The cost‐effectiveness of universal late‐pregnancy screening for macrosomia in nulliparous women: a decision analysis
Source: BJOG. 2019 Jun 5;126(10):1243–50. doi: 10.1111/1471-0528.15809 (PMC6771727; doi:10.1111/1471-0528.15809)
Supplement: Supplementary file 4 — Table S4. Expected costs and QALYs per screening and management strategy, alternative scenarios. [file BJO-126-1243-s004.pdf]

Table S4. Expected costs and QALYs per screening and management strategy, alternative scenarios

Scenario	Policy	Cost (95% CI)	QALY (95% CI)	ICER	Estimated NMB (95% CI)
Base case scenario	Selective US + expectant	2821 (2409, 3236)	27.441 (27.262, 27.621)	-	546007 (542803, 549204)
	**Selective US + induction**	**2826 (2412, 3242)**	**27.446 (27.267, 27.626)**	**904**	**546098 (542890, 549298)**
	Selective US + planned CS	2833 (2436, 3230)	27.417 (27.244, 27.588)	Dominated	545501 (542424, 548561)
	Universal US + expectant	2933 (2502, 3366)	27.441 (27.261, 27.621)	Dominated	545884 (542695, 549070)
	Universal US + induction	2939 (2506, 3374)	27.448 (27.268, 27.628)	52719	546028 (542829, 549214)
	Universal US + planned CS	2955 (2549, 3360)	27.396 (27.224, 27.565)	Dominated	544956 (541919, 547978)
Maternal QALYs excluded from analysis	Selective US + expectant	2821 (2409, 3236)	14.107 (14.069, 14.134)	-	279314 (278388, 280056)
	Selective US + induction	2826 (2412, 3242)	14.111 (14.074, 14.137)	965	279399 (278486, 280125)
	**Selective US + planned CS**	**2833 (2436, 3230)**	**14.115 (14.082, 14.138)**	**1923**	**279463 (278642, 280135)**
	Universal US + expectant	2933 (2502, 3366)	14.106 (14.068, 14.134)	Dominated	279195 (278251, 279955)
	Universal US + induction	2939 (2506, 3360)	14.113 (14.075, 14.139)	Dominated	279328 (278384, 280070)
	Universal US + planned CS	2955 (2549, 3360)	14.119 (14.087, 14.141)	31617.62	279418 (278629, 280072)
No costs for induction of labour	Selective US + expectant	2821 (2409, 3236)	27.441 (27.262, 27.621)	Dominated	546007 (542803, 549204)
	**Selective US + induction**	**2814 (2401, 3230)**	**27.446 (27.267, 27.626)**	**-**	**546110 (542900, 549310)**
	Selective US + planned CS	2833 (2436, 3230)	27.417 (27.244, 27.588)	Dominated	545501 (542424, 548561)
	Universal US + expectant	2933 (2502, 3366)	27.441 (27.261, 27.621)	Dominated	545884 (542695, 549070)
	Universal US + induction	2919 (2486, 3353)	27.448 (27.268, 27.628)	52500	546048 (542850, 549235)
	Universal US + planned CS	2955 (2549, 3360)	27.396 (27.224, 27.565)	Dominated	544956 (541919, 547978)
Induction of labour is cost-saving by £263 (95% CI: -£646, £174).	Selective US + expectant	2821 (2409, 3236)	27.441 (27.262, 27.621)	Dominated	546007 (542803, 549204)
	**Selective US + induction**	**2813 (2400, 3229)**	**27.446 (27.267, 27.626)**	**-**	**546111 (542903, 549311)**
	Selective US + planned CS	2833 (2436, 3230)	27.417 (27.244, 27.588)	Dominated	545501 (542424, 548561)
	Universal US + expectant	2933 (2502, 3366)	27.441 (27.261, 27.621)	Dominated	545884 (542695, 549070)
	Universal US + induction	2917 (2484, 3352)	27.448 (27.268, 27.628)	48321	546050 (542850, 549236)
	Universal US + planned CS	2955 (2549, 3360)	27.396 (27.224, 27.565)	Dominated	544956 (541919, 547978)

Options ordered from lowest to highest expected cost. ICERs calculated beginning with least expensive option, and comparing with next most expensive, non-dominated option; a policy was dominated / extended-dominated if any other policy or weighted average of two policies was associated with both lower costs and higher QALYs. Net monetary benefit (NMB) was calculated using a Willingness-To-Pay (WTP) threshold of £20,000; higher NMB value means greater cost-effectiveness. Option with the highest expected net monetary benefit highlighted in bold. All costs and NMB are given in sterling pounds (£). CS = Caesarean section; QALY = Quality adjusted life-year; ICER = Incremental cost-effectiveness ratio; NMB = Net monetary benefit; US = ultrasound.
